# Supplementary material for: Mechanisms governing the pioneering and redistribution capabilities of the non-classical pioneer PU.1
Source: Nat Commun. 2020 Jan 21;11:402. doi: 10.1038/s41467-019-13960-2 (PMC6972792; doi:10.1038/s41467-019-13960-2)
Supplement: Supplementary file 7 — Source data [file 41467_2019_13960_MOESM7_ESM.zip › Source_Data/Figure5/Figure5A_MotifScanOutput/homerResults/motif47.similar.html]

motif47

## Information for motif47

A
C
G
T
G
A
T
C
A
G
T
C
A
C
T
G
A
C
G
T
A
C
T
G
A
C
T
G
A
C
T
G
A
G
T
C
C
T
A
G
A
C
G
T
C
T
G
A
A
C
T
G
  
Reverse Opposite:  

A
G
T
C
A
G
C
T
C
G
T
A
A
G
T
C
C
T
A
G
A
G
T
C
A
G
T
C
A
G
T
C
C
G
T
A
G
T
A
C
C
T
A
G
C
A
T
G
C
G
T
A
  

|  |  |
| --- | --- |
| p-value: | 1e-33 |
| log p-value: | -7.736e+01 |
| Information Content per bp: | 1.919 |
| Number of Target Sequences with motif | 47.0 |
| Percentage of Target Sequences with motif | 1.56% |
| Number of Background Sequences with motif | 38.4 |
| Percentage of Background Sequences with motif | 0.08% |
| Average Position of motif in Targets | 168.4 +/- 116.5bp |
| Average Position of motif in Background | 187.0 +/- 138.3bp |
| Strand Bias (log2 ratio + to - strand density) | 0.3 |
| Multiplicity (# of sites on avg that occur together) | 1.00 |
| Motif File: | file (matrix) reverse opposite |

### Similar de novo motifs found

|  |  |  |  |  |  |  |  |
| --- | --- | --- | --- | --- | --- | --- | --- |
| Rank | Match Score | Redundant Motif | P-value | log P-value | % of Targets | % of Background | Motif file |
| 1 | 0.910 | T G A C A G T C A C T G A C G T A C T G A C T G A C T G A G T C C T A G A C G T C T G A | 1e-29 | -68.544689 | 1.36% | 0.07% | motif file (matrix) |
| 2 | 0.944 | A C G T G A T C A G T C A C T G A C G T A C T G A C T G A C T G A G T C C T A G A C G T C T G A | 1e-23 | -54.587841 | 1.06% | 0.05% | motif file (matrix) |
| 3 | 0.852 | A C T G A C G T A C T G A C T G A C T G A G T C A C T G A C G T C G T A | 1e-20 | -46.654864 | 1.06% | 0.08% | motif file (matrix) |
